# A review of foot-and-mouth disease in Ethiopia: epidemiological aspects, economic implications, and control strategies

**DOI:** 10.1186/s12985-023-02263-0

**Published:** 2023-12-15

**Authors:** Girma Zewdie, Mirtneh Akalu, Wondwossen Tolossa, Hassen Belay, Getaw Deresse, Mariamawit Zekarias, Yeneneh Tesfaye

**Affiliations:** 1 National Veterinary Institute (NVI), P. O. Box: 19, Bishoftu, Ethiopia; 2 Koneru Lakshmaiah Education Foundation, Department of Biotechnology, Vaddeswaram, Guntur, Ap 522502 India; 3 Africa Union Pan African Veterinary Vaccine Center (AU-PANVAC), P. O. Box: 1746, Bishoftu, Ethiopia

**Keywords:** Control, Diagnosis, Economic importance, Epidemiology, Ethiopia, FMD, FMDV, Virus

## Abstract

Foot-and-mouth disease (FMD) is a contagious viral disease that affects the livelihoods and productivity of livestock farmers in endemic regions. It can infect various domestic and wild animals with cloven hooves and is caused by a virus belonging to the genus *Aphthovirus* and family *Picornaviridae*, which has seven different serotypes: A, O, C, SAT1, SAT2, SAT3, and Asia-1. This paper aims to provide a comprehensive overview of the molecular epidemiology, economic impact, diagnosis, and control measures of FMD in Ethiopia in comparison with the global situation. The genetic and antigenic diversity of FMD viruses requires a thorough understanding for developing and applying effective control strategies in endemic areas. FMD has direct and indirect economic consequences on animal production. In Ethiopia, FMD outbreaks have led to millions of USD losses due to the restriction or rejection of livestock products in the international market. Therefore, in endemic areas, disease control depends on vaccinations to prevent animals from developing clinical disease. However, in Ethiopia, due to the presence of diverse antigenic serotypes of FMD viruses, regular and extensive molecular investigation of new field isolates is necessary to perform vaccine-matching studies to evaluate the protective potential of the vaccine strain in the country.

## Introduction

Ethiopia has a large and diverse livestock population that contributes significantly to the national economy and the livelihoods of rural communities [1]. However, the livestock sector faces major challenges from transboundary animal diseases such as foot-and-mouth disease (FMD), which is a highly contagious viral disease that affects domestic and wild cloven-hoofed animals [2]. FMD causes severe economic losses due to reduced animal productivity, trade restrictions, and control costs. FMD is caused by a virus with seven serotypes (O, A, C, SAT 1, SAT 2, SAT 3, and Asia 1) [3] that vary in their genetic and antigenic characteristics [4]. The virus can infect cattle, sheep, goats, pigs, camels, and several wildlife species [5], causing fever, vesicles, erosions, salivation, lameness, and sometimes death [6].

The disease can spread rapidly through direct or indirect contact with infected animals or materials or through the air [4]. It is common in Africa, the Middle East and Asia, and some parts of South America. FMD-free countries without vaccination are always at risk of outbreaks. FMD prevention and control are costly and mostly borne by low- and lower middle-income countries [7]. The development of novel FMD control measures requires a better understanding of the molecular mechanisms of FMD virus replication and evolution [8]. In Ethiopia, FMD is a notifiable disease that has been reported since 1957 [9]. The disease affects cattle of all ages and breeds, as well as small ruminants and wildlife. The seroprevalence of FMD in Ethiopia ranges from 5.6 to 72.1% in cattle [10, 11] and from 4 to 11% in small ruminants [12]. Four serotypes (O, A, SAT 1, and SAT 2) are endemic in Ethiopia, with serotype O being the most dominant and widespread [13].

The epidemiology and evolution of FMD virus strains circulating in Ethiopia are poorly understood, limiting the effectiveness of control and prevention measures [11]. FMD outbreaks occur frequently across the country, with higher frequency and intensity during the dry season [14]. The occurrence and distribution of FMD outbreaks are influenced by various factors, such as production system, geographic location, species, age of animals, contact with wildlife, season of the year, mixed animal species, breed, and agroecology [15, 16].

New FMDV topotypes in Ethiopia challenge the livestock industry and need better understanding of the disease and its serotypes. This will help design effective control and prevention measures such as vaccination, rapid diagnostic tests (RDTs), and early warning systems [17]. FMD has significant impacts on livestock production and trade in Ethiopia due to reduced animal performance and welfare, market access barriers and consumer confidence, and vaccination costs. Therefore, the aim of this review is to provide a comprehensive overview of FMD with global up-to-date information on its epidemiology and current status in Ethiopia.

## General features of FMD

FMD is a viral disease that affects various cloven-hoofed animals and causes huge economic losses in the livestock sector worldwide. It is caused by a virus from the genus *Aphthovirus* in the family *Picornaviridae*, which has a single-stranded RNA genome of approximately 8.5 kb [18–20]. The genome contains one open reading frame (ORF) surrounded by 5′ and 3′ untranslated regions (UTRs) and a poly-A tail at the 3′ end [21]. These regions play important roles in FMDV replication and translation by affecting viral RNA stability, translation efficiency, genome circularization, and interaction with cellular factors [22]. The RNA has two parts that are important for this explanation: the 5′UTR and the 3′UTR. The 5′UTR helps the virus to start making proteins, while the 3′UTR helps the virus to control how much protein is made. The 5′UTR and the 3′UTR can also interact with each other to regulate protein expression [23]. The poly-A tail of viral RNA interacts with cellular factors that affect its stability, translation initiation, and genome synthesis. These factors include Poly (A) binding proteins (PABPs), Exoribonucleases (ExoNs), and Poly (A) polymerases (PAPs). PABPs regulate the poly-A tail function and fate by modulating its recognition by host pattern recognition receptors (PRRs) and signaling molecules. ExoNs degrade RNA from the 3’ end and can alter the length and function of the poly-A tail. PAPs synthesize the poly-A tail by adding adenine nucleotides to the 3’ end of RNA and can influence the replication, transcription, and translation of viral RNA. The untranslated regions (UTRs) and the poly-A tail are essential for foot-and-mouth disease virus (FMDV) infection and pathogenesis [23–25, 31–33].

All the seven FMDV serotypes are differ in their antigenicity, virulence, and host range [34]. Antigenicity is the capacity of a molecule or an antigen to induce an immune response, that is to be recognized by and interact with an immunologically specific antibody or T-cell receptor [35]. Antigenicity depends on several factors, such as the antigen processing, presentation, and recognition by antigen-presenting cells (APCs), the B-cell receptor (BCR) epitope interactions, and the affinity and avidity of the antibody-antigen binding. Antigenicity also determines the memory and protection of the immune system on re-encounter with the same antigen [36]. Serotype C has not been detected since 2004. The VP1 protein of FMDV contains the major B-cell epitope, which is the part of the antigen that is recognized by antibodies and induces protective immunity. VP1 also shows high variability and phylogenetic clustering among FMDV strains. Therefore, sequencing and classification of FMDV strains are based on the VP1 gene, which helps to track their evolution and spread [4, 26–30].

**Fig. 1:**
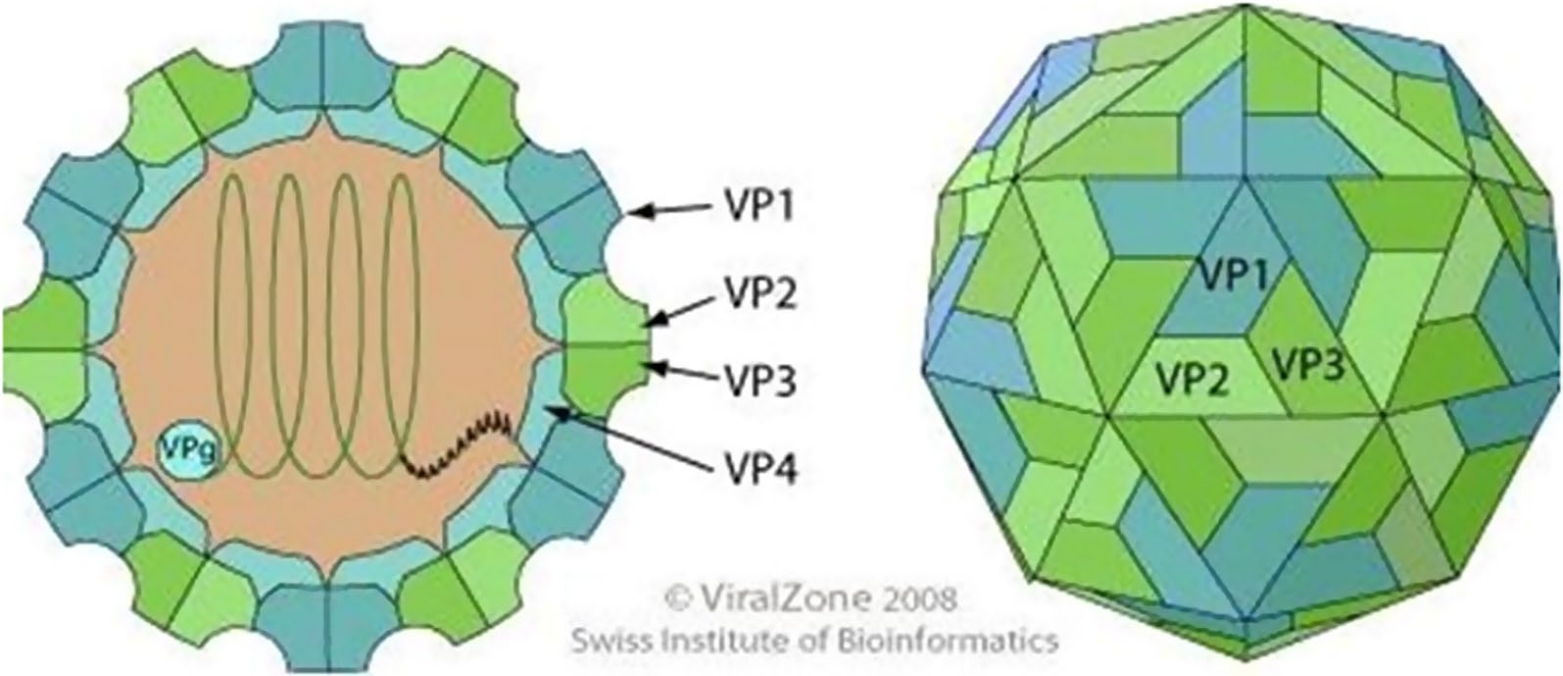
FMDV structure (icosahedral capsid structure), adapted from https://viralzone.expasy.org/98. (Accessed on 17 July 2022)

The capsid of FMDV is made up of 60 copies of four structural proteins, called VP1, VP2, VP3, and VP4. These proteins are also known as 1A-1D. The capsid has a symmetrical shape with 20 triangular faces (Fig. 1) [37]. The capsid has an icosahedral shape with 20 faces that are each triangular. The proteins VP1, VP2, and VP3 are exposed on the capsid surface, while VP4 is internal and contributes to the structural integrity. The capsid proteins originate from a precursor protein called P1-2A, which is cleaved by a viral protease named 3Cpro into smaller fragments [37, 38]. The capsid proteins can self-assemble into empty shells that resemble the virus but lack any genetic material. These empty shells can serve as vaccines to induce immunity against FMDV in animals [37].

VP-1 is a multifunctional structural protein that mediates host-cell attachment and elicits immune responses and cell apoptosis [39]. It is the most variable region of the capsid gene and contains highly immunogenic and neutralizing sites [38]. Viral protein 2 (VP2) is a structural protein that contributes to the stability and maturation of the FMDV capsid. It is relatively conserved among FMDV serotypes, with 47% of its amino acids being identical across and within serotypes [20]. However, the extent of conservation varies depending on the region of VP2. The N-terminal end is highly conserved, with 67.4% invariant amino acids, while the C-terminal end is more variable. VP2 also has some antigenic and surface-exposed sites that are conserved among serotypes and may be important for vaccine development [40].

The capsid is composed of four structural proteins: VP1, VP2, VP3, and VP4. Among them, viral protein-1 (VP1) is the most important (major antigenic site of FMDV) for determining the serotype and the immune response of the host. VP4 is the most conserved protein (nucleotide sequence that is 73% to 84% conserved across all FMDV isolates) and has a T-cell epitope for swine [20]. The variability of the capsid proteins is influenced by selective pressures from the host immune system, cell culture adaptation, and vaccine escape [41]. The antigenic sites of FMDV are located on the surface-exposed loops of VP1-3, especially the βG–βH loop in VP1 [42].

**Fig. 2:**
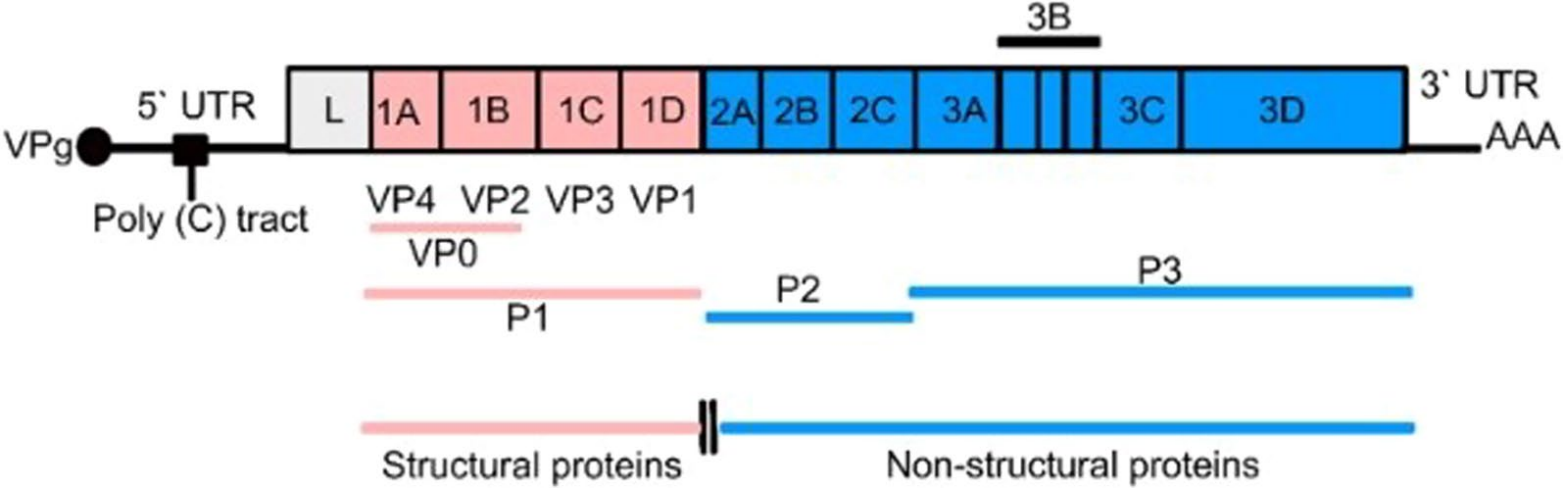
The RNA strand of FMDV and its protein-coding parts. The RNA strand has a 5′UTR, an ORF with L, VP4, VP2, VP3, VP1, 2A, 2B, 2C, 3A, 3B (3B1, 3B2, and 3B3), 3C, and 3D parts, and a 3′UTR.; source: [25]

The open reading frame (ORF) encodes four structural proteins (VP1, VP2, VP3, and VP4) that form the viral capsid and eight nonstructural proteins (NSPs) (Lpro, 2A, 2B, 2C, 3A, 3B, 3Cpro, and 3Dpol) that help the virus replicate and evade host defenses [25] (Fig. 2). These viral proteins play various roles in FMDV pathogenicity and can inhibit the functions of various host proteins to counteract host antiviral responses [39]. The mutation of the H138 residue in Lpro, an NSP that inhibits host translation, reduces FMDV virulence in vitro and in vivo [43].

Leader proteinase (Lpro) is the first mature viral protein in FMDV and a key virulence factor that inhibits host cell translation. FMDV Lpro is a viral enzyme that belongs to the papain-like protease family. It cuts the viral polyprotein into smaller proteins and targets some host proteins. This affects the ability of the host cell to make its own proteins and activate its immune system against the virus [43]. FMDV has a genome composed of a single strand of RNA without an envelope surrounded by a protein shell that is not symmetrical [44]. The virus makes mistakes when copying its RNA, leading to high variation among the different serotypes of the virus, which have approximately 86% similarity and cause repeated outbreaks of the disease [45]. Therefore, FMDV serotypes comprise antigenically distinct strains and topotypes, leading to partial cross-protection within serotypes [46].

### Morbidity and mortality rate

FMDV is a highly contagious virus that causes FMD, a viral disease that infects various cloven-hoofed animals. FMD can impair the health and productivity of these animals and result in huge economic losses worldwide. The disease is endemic in more than 100 countries, mostly in Africa, Asia, and South America [2]. FMD is classified as the most dangerous animal disease on list A by the World Organization for Animal Health (WOAH), but it does not affect humans. The disease has an incubation period of 2–14 days. The disease is rarely fatal in adult animals (1–5%), but high in young calves, lambs, and piglets (20% or more) [47]. However, the morbidity and mortality rates of FMDV depend on various factors, such as the animal species, breed, production type, age, immunity, virus dose, and animal movement [48]. In a fully susceptible livestock population, the morbidity rate of FMDV can be as high as 100% with a high mortality rate in young animals due to myocarditis [47]. In Ethiopia, FMDV is endemic, with different serotypes circulating at different prevalence levels [2].

The disease impairs the performance and productivity of livestock, especially cattle, which are vital for many farmers' income and food security. A recent study found that FMDV morbidity rates in cattle herds were 68.1% in mixed crop-livestock systems and 54.5% in commercial dairy farms. Another study reported a lower rate of 38.9% during major outbreaks [49]. FMDV infection causes low mortality (0.4%) in cattle and high morbidity (35.7%) in sheep and goats, which can also be asymptomatic carriers of the virus. The herd-level morbidity of FMDV is higher in cattle (57.2%) than in small ruminants (8%) in mixed systems [49]. Most animals recover from FMDV infection within 2 weeks if there are no complications [47]. Sheep and goats are less susceptible to FMDV infection than cattle, but they can still transmit the virus in mixed farming systems [50].

### Pathogenesis of FMD in animals

FMDV infection causes different clinical signs, pathogenesis, and immune responses depending on the host and serotype [51]. The virus can enter the host cell by binding to cellular receptors through its capsid proteins. The virus makes copies of itself in the pharyngeal mucosa and travels through the lymphatic and blood systems to other epithelial tissues in the mouth, feet, mammary glands, and skin [52]. The virus can be found in various body fluids before and after the symptoms of FMD appear. The virus can also stay in the oral cavity of infected animals for a long time. The host antiviral system quickly responds to stop virus replication and clear the virus. However, viruses evade host antiviral responses and affect host resistance to other pathogens [34].

FMDV has evolved different immune escape mechanisms to overcome the host immune response in long-term coevolution. These mechanisms can be classified into three types: (1) changing or masking the antigenic structure of the viral surface to avoid recognition by the humoral immune response; (2) inhibiting the expression or activity of cytokines, chemokines, or major histocompatibility complex (MHC) molecules to interfere with the function of the cellular immune response; and (3) inducing immunosuppression, apoptosis, or tolerance to interfere with the host’s immune response to the virus [53].

## The epidemiological features of FMDV

### Host range

Foot-and-mouth disease virus (FMDV) is a virus that causes FMD, a viral disease that affects animals with split hooves, such as cattle, sheep, goats, pigs, and many wild ruminants. Cattle are the main hosts for FMDV and the most vulnerable to infection. However, some FMDV strains (serotype O) can adapt to pigs after being isolated in cell culture. The role of small ruminants in FMDV transmission is unclear, as it is unknown whether the virus can stay in these animals for long periods without infecting cattle. However, FMDV strains that infect cattle have also been found in wild pigs, antelopes, and deer [54]. Therefore, these hosts have clinical and epidemiological importance in natural infection. Other susceptible species (deer, antelope, wild pigs, elephants, and giraffes) are infected incidentally or accidentally and have little or no epidemiological significance [55]. Horses are resistant to FMD, but the mechanism of resistance is unknown [56]. FMD does not harm human health or food safety, but only healthy animals should be used for consumption [57]. However, FMD can devastate the livelihoods and food security of poor communities, as it reduces animal productivity and income [58].

### FMDV transmission and risk factors across species and regions

FMD is mainly transmitted by inhaling virus particles from the breath of infected animals. However, the disease can also spread through the air under certain conditions. The virus can travel through the air under favorable conditions and infect animals outside quarantine zones, making control measures difficult [59]. Cattle are mostly infected by the respiratory or airborne route, while pigs can be infected by the digestive route through contaminated food, water, or fomites [60].

The virus can also spread indirectly through the environment if it is contaminated by infected animals. The virus can survive for a long time under favorable conditions, such as temperatures below 50 °C, relative humidity above 55%, and neutral pH [61, 62]. This makes FMD epidemiology and control more complex, as the virus can have multiple sources of infection. The excretions and secretions of infected animals can transmit the disease and serve as noninvasive samples for diagnosis and surveillance [62].

The occurrence of FMD outbreaks in Ethiopia is influenced by various factors, such as production system, geographic location, species, age of animals, contact with wildlife, season of the year, mixed animal species, and breed [2, 17]. FMD outbreaks are more prevalent in market-oriented systems than in subsistence systems due to frequent movement and mixing of animals [63]. The central, southern, and southeastern regions have a higher incidence of outbreaks than the northern and western regions, which may be related to wildlife reservoirs, climatic factors, or livestock density [63]. Younger animals are more prone to FMD infection than older animals, as they have lower immunity and higher virus shedding. They also tend to show more severe clinical signs and higher mortality rates [12, 14].

Contact with wildlife is another risk factor for FMD occurrence [15], although the only confirmed wildlife reservoir is the African buffalo, S*yncerus caffer* [7]. Wildlife can also be affected by FMD outbreaks and act as bridge or maintenance hosts for disease transmission. Livestock and wildlife can come into contact by sharing the same grazing or watering areas or breaking through fences [12, 64]. This increases the risk of FMD outbreaks, especially in the dry season when water and pasture are scarce. The virus can also survive better in the environment during the dry season [14].

Keeping different species of livestock together can increase the risk of FMD transmission, as different species may have different susceptibilities and immunity to the virus. For example, sheep and goats can act as silent carriers of FMD and infect cattle without showing clinical signs. Some breeds of cattle are more resistant to FMD than others due to genetic factors that modulate their immune response to the virus. For instance, Borana cattle in Ethiopia are less likely to become infected and show less severe symptoms than other breeds [14]. FMD virus poses challenges for diagnosis, surveillance, and vaccination because it has many genetic variants. The disease also affects the livelihoods and incomes of livestock farmers and traders in developing countries, where livestock are important for food security and social status. Therefore, the socioeconomic factors that influence the attitudes and knowledge of livestock stakeholders are crucial for preventing and controlling FMD.

### Historical background

Frosch and Loeffler discovered FMDV as the cause of FMD in 1897, paving the way for virology. Fracastorius described the symptoms of FMD in cattle in 1514 [65]. His description matched the disease seen in Germany (1751) and the UK (1839). The disease spread beyond New Zealand and is still common in parts of Asia, Africa, and South America [66]. The virus also diversified into seven serotypes and originated from the Mediterranean region before reaching Europe, Asia, and South America [27, 67].

### Global distribution

FMDV may have emerged in the Middle Ages and spread from the Mediterranean area to other parts of the world through trade and migration. FMDV can survive for long periods in favorable conditions. FMDV has seven serotypes that vary in their molecular characteristics and evolutionary relationships. FMDV also undergoes frequent recombination events that increase its genetic variation and pose challenges for vaccine development and selection [68]. Most of the World’s livestock (77%) is affected by FMD, especially in Africa, the Middle East, Asia, and some parts of South America. The disease can spread to countries that are FMD-free despite vaccination, as some vaccinated animals may still be susceptible to infection or become carriers of the virus. For example, in 2019, an outbreak of FMD occurred in Mongolia, which had been FMD-free since 2017 and had implemented a vaccination program. The outbreak was caused by a new strain of FMDV that was not covered by the vaccine [7]. FMD prevention and control costs are mostly (75%) borne by low- and lower-middle-income countries. These costs are highest in Africa and Eurasia, which account for half and a third of the global total, respectively [7].

The official data show that China has experienced 140 FMD outbreaks since 2010. China has reported a total of 109 FMD outbreaks from 2010 to 2019, affecting cattle, pigs, sheep and goats in 24 provinces [69, 70]. The most recent event occurred in October 2020, affecting 76 cattle in Heshuo County, Xinjiang Uyghur Autonomous Region [71]. Moreover, the common practice of small-scale farming and grazing in these regions could also increase the risk of FMD transmission [71].

Brazil and Kenya are the last countries where serotype C of FMD virus was detected in 2004. Serotype C has been considered extinct since then [65]. The United Kingdom experienced a major FMD outbreak in 2001 that resulted in the slaughter of over six million animals and an estimated cost of £8 billion [72]. India is one of the countries with the highest economic impact of FMD due to its large livestock population and low vaccination coverage. The annual losses due to FMD in India are estimated at USD$4.5 billion [73].

**Fig. 3:**
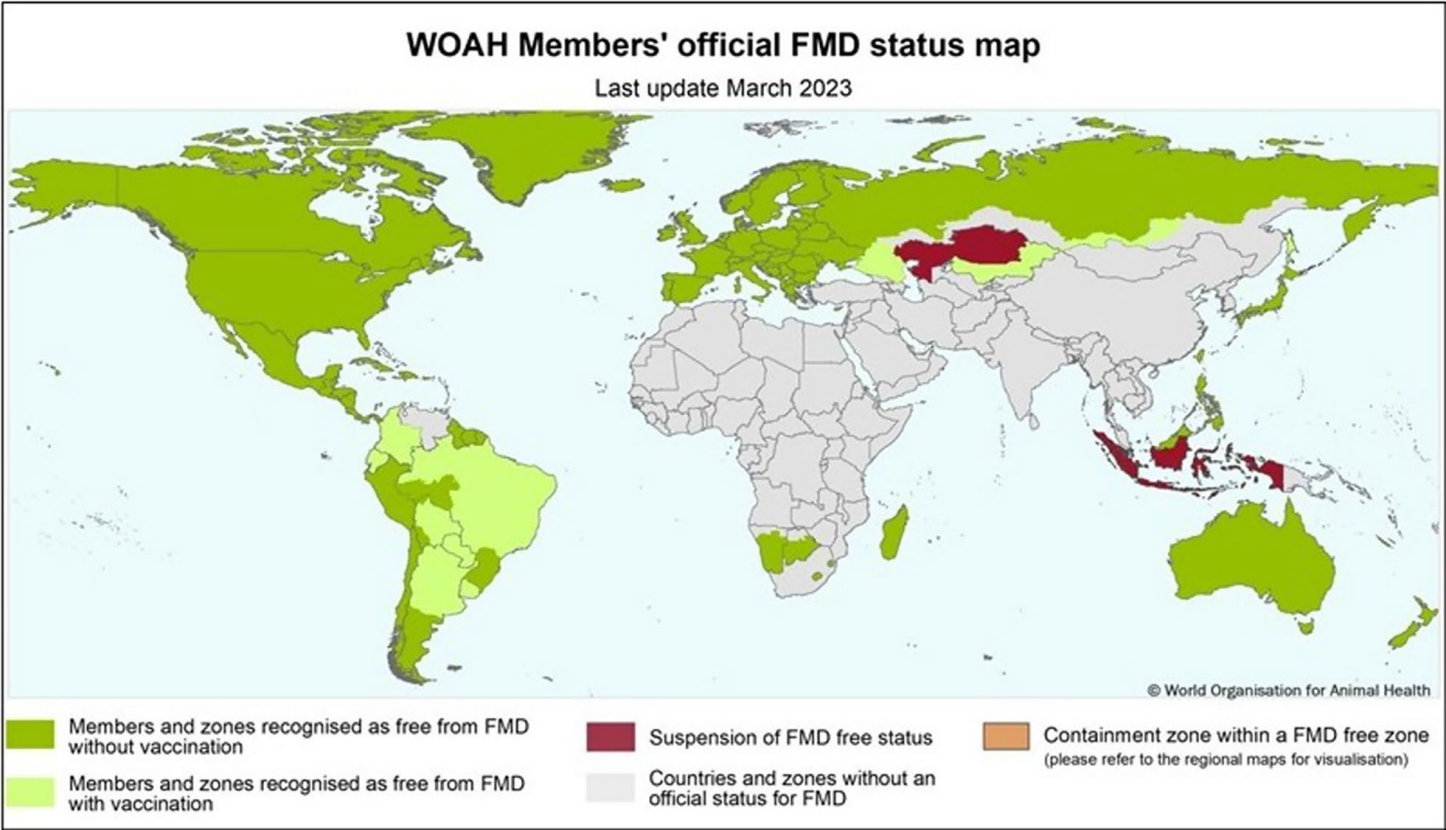
Map of FMD official status; adapted from https://www.woah.org/app/uploads/2023/03/fmd-world-eng-2023.jpg (accessed on 17 July 2022)

In 2012, the FAO and OIE launched a 15-year global strategy to control FMD. The strategy aims to mitigate the adverse effects of FMD on animal health and welfare, food security and economic development. The strategy involves enhancing the capabilities of veterinary services and adopting progressive control measures informed by risk assessment and surveillance [74]. Early detection, warning systems and surveillance are crucial for preventing the disease. The WOAH maintains an official list of FMD-free countries that can be recognized as free of the disease in their whole territory or in specific zones and compartments (Fig. 3). FMD is a viral disease of livestock that can spread across national borders and infect many animals. Most countries in Latin America have achieved FMD-free status by implementing zoning and vaccination strategies. FMD is absent from some other regions of the world, but it can still emerge sporadically [7].

**Fig. 4:**
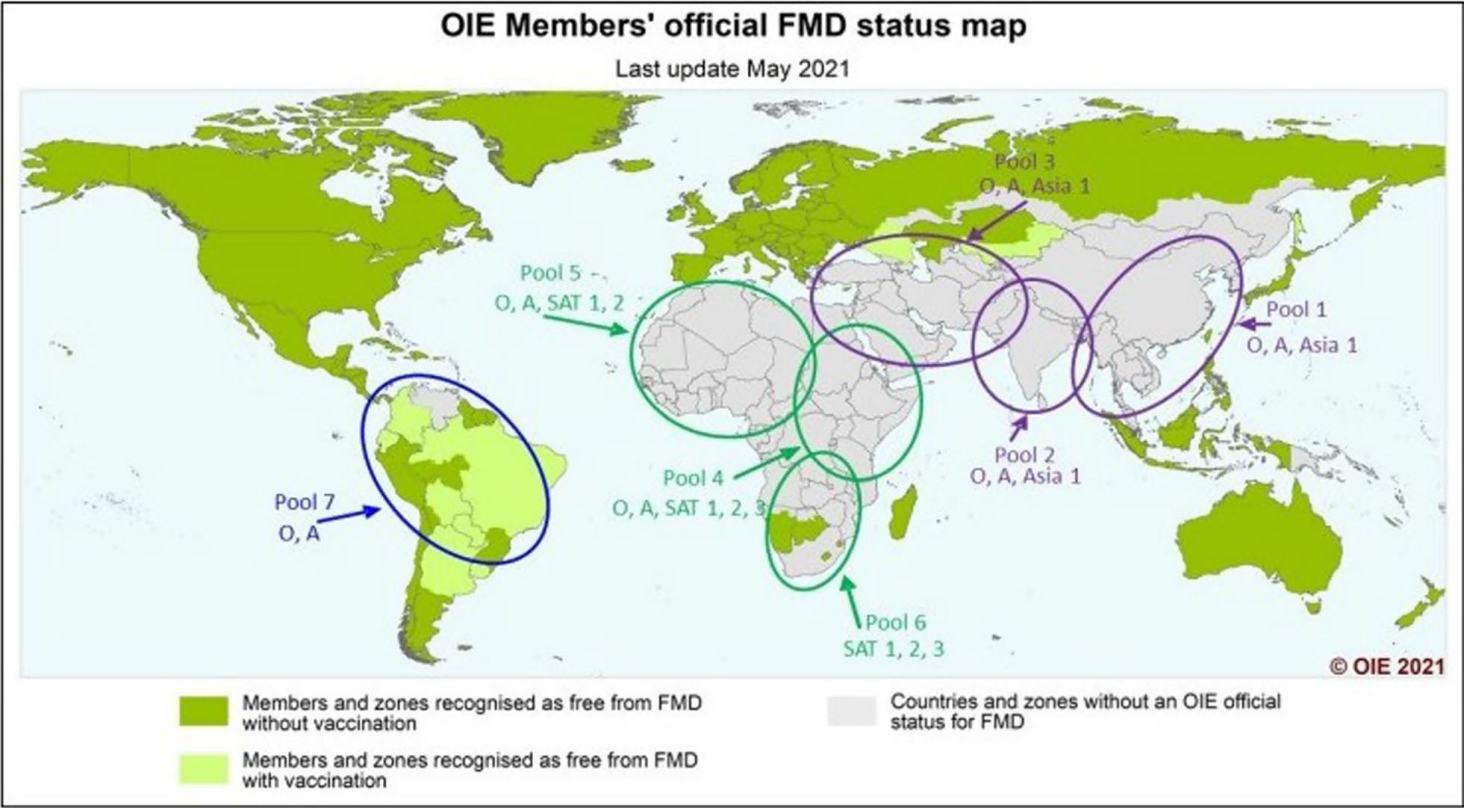
Endemic serotypes of FMDV in different pools; adapted from https://www.woah.org/app/uploads/2021/05/fmd-world-eng.png (accessed on 17 August 2023). Source [68]

FMDV has seven serotypes: O, A, C, Asia 1, SAT 1, SAT 2, and SAT 3. These serotypes have different sub-lineages or topotypes based on the genetic and antigenic diversity of the capsid genes, especially the VP1 region. The VP1 region can vary by 30–50% between serotypes, while the overall similarity between serotypes is approximately 86%. FMDV is constantly evolving in the field, producing new strains that can cause outbreaks and spread to new areas. FMDV serotypes and their sub-lineages or topotypes are classified into seven virus pools based on their geographic and characteristic features (Fig. 4) [68]. These pools are distributed across Europe, the Middle East, Asia, Africa, and the Americas [68, 80].

Viral protein1 (VP1) is the most important structural protein of FMDV, as it determines the serotype and the immune response of the host. VP1 forms part of the capsid, the outer layer of the virus that protects its genetic material and interacts with the host cell receptors. VP1 is the most exposed and variable protein on the surface of the capsid, and it contains the major antigenic site of FMDV, which is recognized by the host’s antibodies. VP1 is also involved in the attachment of FMDV to the host cell receptors, such as integrins and heparan sulfate proteoglycans. VP1 can change its shape and antigenicity to escape from the host’s immune response and infect new cells [77, 78].

Viral protein1 (VP1) is the most important target for vaccine development and selection against FMDV. Vaccines are substances that stimulate the immune system to produce antibodies against a specific pathogen, without causing the disease. Vaccines can protect the host from future infections by the same or similar strains of the pathogen. However, due to the high variability of FMDV, especially in the VP1 region, the vaccines need to match the circulating strains of the virus to be effective. Therefore, it is essential to monitor the evolution and circulation of FMDV serotypes and their sub-lineages or topotypes and to update the vaccines accordingly [79].

FMDV serotypes vary in their geographic distribution and impact. Serotypes O, A, and C can cause outbreaks in Europe, America, Africa, and Asia. Serotypes SAT 1–3 and Asia-1 are restricted to Africa and Asia, respectively [75]. Serotype O is the most common in Africa, except for Southern Africa. Serotype O is also the dominant serotype in Ethiopia [76]. Serotype C has not been detected since 2004 in Brazil and Kenya and is considered extinct [27]. Therefore, the evolution and circulation of viruses within different geographic pools help to assess the suitability of vaccines [17].

### Current status of FMD in Ethiopia

FMD was first detected in Ethiopia in 1957, when serotypes O and C were isolated from cattle [9]. Since then, other serotypes, such as A, SAT 1, and SAT 2, have been reported in different regions of the country, with serotype O being the most dominant and serotype C being absent since 1983 [3, 81]. FMD is endemic and widely prevalent in Ethiopia, affecting various livestock species and causing economic losses [82]. A recent study conducted by Woldemariyam, et al. [83] revealed the spatiotemporal trends and hotspots of FMD outbreaks in Ethiopia over 10 years. They observed that the dry season (October–March) was associated with a higher frequency and intensity of outbreaks, with a peak in March 2012. They also noted a decreasing trend in outbreaks over time, but with a potential duration of up to 5 years. They identified four dominant serotypes (O, A, SAT-2, and SAT-1), but only 12% of outbreaks had a known serotype. According to Ayelet, et al. [13], FMD was more prevalent in the central and southern parts of Ethiopia, indicating that the disease was endemic and had multiple serotypes.

[84] reported that serotypes A and SAT 2 were first detected in 1969 and 1989, respectively, and that serotypes O and SAT 2 caused an outbreak between 1988 and 1991. Only one outbreak of serotype SAT 1 was studied from 2007 to 2008 [85]. Asfaw and Sintaro [86] noted that FMD outbreaks had become more frequent since 1990 and were not effectively controlled due to the lack of regular vaccination, except for some dairy animals. This led to substantial economic losses for farmers and the country due to trade restrictions on livestock and livestock products [87]. FMD control in Ethiopia faces major challenges due to the presence of multiple serotypes and subtypes of the virus, the diversity of host animals, the unregulated movement of livestock, and the shortage of effective and affordable FMD vaccines in the country. Therefore, a long-term progressive risk reduction approach for FMD control is recommended for endemic countries such as Ethiopia [79, 88].

#### Sero-prevalence of FMD

Table 1 shows the FMD seroprevalence in Ethiopia from 2008 to 2021 based on different studies. The FMD seroprevalence in Ethiopia varies by location, from 5.6% to 42.7% in cattle, 4% to 11% in small ruminants, and 30% in ungulate wildlife [12]. In the Borena zone, 42.7% of 363 cattle sera samples were positive for FMD antibody using the 3ABC-ELISA test [82]. The highest prevalence was in Dire district (52.8%). However, in North and South Gondar, the FMD seroprevalence in cattle was lower (14.9%) [89]. This could be due to more FMD outbreaks during a period of major socioeconomic and political crisis (Guerrini et al., 2019). In the Afar region, Dubie and Negash [15] found that 19.8% of animals and 56.94% of herds were FMD seropositive, based on 384 sera samples. Shurbe, et al. [16] also reported that 26.8% of cattle in the Gamo zone had FMD antibodies. In northern Amhara, the FMD seroprevalence in cattle was low (3.4%) [90], which was similar to the 3.1% reported by Gezahegn, et al. [91].

**Table 1:** FMD seroprevalence in cattle reported in Ethiopia between 2008 and 2021

Authors	Study duration	Location	Total Sample	Reported
				prevalence %
[96]	2008–2009	South Omo Zone		
		Gnangatom	61	13.1
		Jinka	162	4.9
		Hammer	104	13.5
		Malle	123	3.3
		Semen Aari	142	2.8
		Bennatsemay	94	20.2
		Dasanech	84	7.1
[97]	2010–2011	Dire Dawa	752	8.91
		East Harerge	234	5.13
[10]	2011	Kellem Wollega		
		Sayo	111	31.53
		Dale Sadi	131	15.26
		Lalo Kile	142	19.01
[98]	2014–2015	East Shewa (Oromia)	69	10.88
[82]	2015	Oromia		
		Dire	125	47.2
		Moyale	68	29.4
		Yabello	170	40.6
[99]	2016	Adama	190	26.8
		Asella	384	22.9
[89]	2016	North Gondar	370	17.8
		South Gondar	208	9.6
[94]	2017–2019	Oromia (west Shewa)		
		Ambo	66	28.8
		Bako Tibe	78	38.5
		Cheliya	50	56
		Abuma Gindeberet	51	47.1
		Jeldu	43	30.2
		Tokekutaye	96	42.7
[15]	2018–2019	Asayita	13	48.13
		Dubti	17	85
		Chifra	11	44
[11]	2019–2021	Ada Berga	105	97.2
		Holeta	65	71.4
		Sululta	106	57.6
[16]	2021	Gamo zone		
		Lowland	–	64.57
		Midland	–	9.3
		Highland	–	5.88

According to Bahiru and Assefa [90], a study conducted in northern Amhara, Ethiopia, found that the overall seroprevalence of FMD was 3.4%, with no significant association with risk factors such as age, sex, district, and body condition score of the cattle. The prevalence was lower than other parts of the country. This result was much lower than the 10.88% prevalence reported in Jijiga [92] and the Amhara region [93], which could be due to different animal management practices, study years, and environmental factors [90].

A cross-sectional study around Addis Ababa found that the FMD seroprevalence among dairy cattle was high (72.1%). The highest seroprevalence was in the Ada Berga, Holeta, and Sululta districts (97.2%, 71.4%, and 57.6%, respectively). This study also found some risk factors for FMD that were statistically significant and detected FMDV serotype O from outbreak cases [11]. However, lower seroprevalence was reported in the West Shewa Zone (40.4%) [94] and in feedlot cattle in Meki (Oromia) (37.4%) [95].

The prevalence of FMD varies by region and country due to different factors, such as sampling method, study design, virus characteristics, and local conditions [16]. Some of these factors are agroecology, wildlife contact, animal movement, grazing and watering practices, age, body condition, management system, vaccination status, vaccine quality, diagnostic capacity, and livestock species interaction [15, 16]. Awel, et al. [11] also reported that age, body condition, and management system of the animals affected FMD seroprevalence in Ethiopia. They suggested that the high seroprevalence in some areas could be related to the diversity of FMDV subtypes and/or topotypes and the low FMD vaccination coverage in those areas.

Several studies have also reported differences in FMD seroprevalence among cattle based on sex, age, breed, and agroecological zone. Jenbere, et al. [100] reported higher seroconversion rates in male (15.7%) than female (8.3%) animals, while Mesfine, et al. [101] reported the opposite trend (8.9% in females and 3.0% in males). Desissa, et al. [10] reported higher FMD seroprevalence in female (27.17%) than male (15.34%) animals in the Kellem Wollega Zone of Ethiopia. They also noted that older cattle (> 4 years) had the highest seroprevalence (24.22%), followed by middle-aged (2–4 years) and young (< 2 years) cattle. Shurbe, et al. [16] reported that in the Gomo zone of southern Ethiopia, FMD seropositivity was higher among adult, local, and lowland cattle than among young, crossbred, and highland cattle. However, this finding was contradicted by G, et al. [102], who asserted that crossbred and productive cattle were more susceptible to FMD than local cattle. They recommended restricting animal movement and contact to reduce FMDV antibody levels in stationary systems [103].FMD seroprevalence in Ethiopia was lower than in other countries in the region, such as Kenya and Eritrea, where higher rates were observed using different diagnostic methods [104–106]. They suggested conducting more comprehensive and coordinated seroprevalence studies in Ethiopia to elucidate the molecular epidemiology of FMDV and devise effective control strategies. FMD outbreaks in Ethiopia have increased over time, especially in the Oromia and Amhara regions, where the highest number of outbreaks occurred in 2004, 2007/2008, and 2011/2012 [13, 17, 103]. The high FMD incidence in some regions may be attributed to various factors, such as susceptible animals, animal movement, market contact, and shared resources [76].

#### Risk factors associated with FMD infection and transmission

FMD infection and transmission can be affected by various factors related to the virus, the host, and the environment. Viral factors include the serotype, genotype, virulence, antigenic variation, and persistence of FMDV in the environment or in carrier animals. Host factors include the species, breed, age, immune status, susceptibility, and density of susceptible animals [7, 107]. The environmental factors include the climate, season, geography, vegetation, wildlife reservoirs, and human activities. Some of the human activities that can raise the risk of FMD infection and transmission are unregulated or illicit movement of animals or animal products across borders or within regions [108, 109]. Mixing of animals at water points, grazing areas, markets, or slaughterhouses; contact with infected wild animals or their products; insufficient biosecurity measures at farms, transport vehicles, or slaughter facilities; and low effectiveness or coverage of vaccination programs [107, 109].

Factors such as production system, geographic location, wildlife contact, seasonality, animal species, and breed influence FMD outbreaks in Ethiopia [2, 17]. FMD incidence is higher in market-oriented systems and central, southern, and southeastern regions than in subsistence systems and northern and western regions [63]. Young animals are more susceptible to FMD than older animals [12, 14]. Wildlife contact can occur through shared resources or fence breaking and can increase FMD transmission risk. The African buffalo, *Syncerus caffer,* is the only confirmed wildlife reservoir of FMD [7]. FMD outbreaks are more frequent during the dry season than during the wet season [14]. Mixing of different livestock species can enhance FMD transmission [15], as they may have different levels of susceptibility and immunity to the virus. FMD resistance varies among cattle breeds [14]. The genetic diversity of FMDV challenges its diagnosis, surveillance, and vaccination. FMD also has severe socioeconomic impacts on livestock stakeholders in developing countries. Socioeconomic factors affect the prevention and control of FMD by influencing the behavior and awareness of livestock owners and traders.

### Molecular epidemiology of FMDV

Molecular epidemiology is essential for understanding FMDV distribution and disease situation and for developing effective control strategies. It uses genetic sequences of FMDV strains, particularly the VP1 gene that encodes the major capsid protein and antigenic determinant of the virus, to infer their origin, spread, and relatedness. VP1 can also help trace virus diffusion associated with animal movements, interspecies transmission events, transcontinental introductions, and the spatiotemporal distribution of viruses involved in FMD outbreaks [110]. However, whole-genome sequencing of FMDVs that cause subclinical infections has been reported in few studies [111], which is a better tool for tracing transmission within and between herds [112].

Molecular epidemiology can also track the emergence and distribution of new FMDV variants that may threaten animal health and trade [26, 113]. The main antigenic sites of the FMDV capsid have been characterized to trace virus strains and their transmission patterns [113]. FMD occurrences are also influenced by interspecies transmission, livestock movements (both formal and informal), and geographic relatedness among FMD virus isolates. Whole genome sequencing can help distinguish between closely related viruses and reconstruct transmission pathways between farms within outbreaks [114]. The high rate of replication errors in the FMD viral RNA replication process leads to high genetic diversity among virus serotypes, which share approximately 86% homology [115]. VP1 is a variable protein that contains the major immunogenic epitopes of FMDV. It varies by 30–50% among the seven serotypes. It is important for FMDV molecular epidemiology research worldwide [110].

Phylogenetic analyses of VP1 nucleotide sequences can reveal variation, genetic relationships, and geographical distributions among different FMDV serotypes [116]. Therefore, the VP1 genomic region is useful for molecular diversity analyses that determine lineages and topotypes. However, full genome sequences can provide more insights into the evolution and virulence of cocirculating strains [117], as they have serotype-specific amino acids that allow for serotype differentiation [118]. The complete genome sequence of FMDV, especially the ORF region, can reveal the amino acid changes that affect the serotype-specific immunogenicity, antigenicity, disease outcome, and transmission of the virus [119]. Similarly, molecular analysis of FMDV capsid proteins can provide detailed information on the antigenic and genomic characteristics of the virus serotype [120]. The presence of a high FMD-susceptible population, vaccine- and infection-induced partial antibody responses in some areas, and animal movement facilitate the establishment and spread of FMDV genetic lineages [121].

The VP1 region of the virus is commonly used for phylogenetic analysis to study the origin and spread of FMD in different regions of the world [110]. Understanding the outbreak pattern and epidemiology is essential for effective control strategies [17]*.* In Ethiopia, five FMDV serotypes (except Asia-1 and SAT3) were isolated from 1974 to 2007 [99]. Serotypes A and O are the most common and have a high impact on livestock production in Ethiopia [76]. Full genome sequences can provide more insights into the evolution and virulence of cocirculating strains [117], as they have serotype-specific amino acids that enable serotype differentiation [118]. Molecular analysis of the entire virus genome, especially the ORF region, can detect the amino acid sequence changes that indicate FMDV-specific serotype immunogenicity, antigenicity, disease prognosis, and spread [122].

Moreover, molecular examination of FMDV viral capsid proteins can provide comprehensive data on the antigenic and genomic determinants of the virus serotype [120]. The presence of a high FMD-susceptible population, vaccine- and infection-induced partial antibody responses in some areas, and animal movement facilitate the establishment and spread of FMDV genetic lineages [121]. FMDV serotypes A, O and SAT 2 caused most FMD outbreaks in Ethiopia [99]. However, in 2018, FMD outbreaks caused by serotypes O and A were reported, but genomic analysis showed that serotype A was the predominant serotype in the study areas [3], contrary to previous reports that serotype O was the most common, followed by serotype A [13, 123]. One sample had a coinfection of both serotypes. Serotypes O and A were further classified as East Africa and Africa topotypes of genotype IV, respectively [3].

FMDV serotype O is the most prevalent and widespread serotype in Asia, where it causes frequent outbreaks of FMD in susceptible animals. This serotype has 11 topotypes, which are groups of viruses that share genetic similarities in the VP1 gene. The VP1 gene encodes the major capsid protein and antigenic determinant of the virus. The topotypes are named after their geographic regions: Europe-South America (Euro-SA), Middle East-South Asia (ME-SA), Southeast Asia (SEA), Cathay (CHY), West Africa (WA), East Africa 1 (EA-1), East Africa 2 (EA-2), East Africa 3 (EA-3), East Africa 4 (EA-4), Indonesia-1 (ISA-1), and Indonesia-2 (ISA-2) [124, 125].

FMDV serotype Asia 1 has seven genotypes: I to VII. The topotypes and genotypes differ in their geographic distribution, antigenicity, virulence, and host range. In Asia, the dominant topotypes of FMDV serotype O are CHY, ME-SA, and SEA, while the dominant topotype of FMDV serotype Asia 1 is Cathay [126]. The ME-SA topotype viruses were mainly found in Egypt and Libya, reflecting their trade links with the Middle East [125]. The EA topotypes (EA-1 to EA-4) are dominant in Africa. Serotypes A and C were grouped into their African topotypes. Type A was similar to the serotypes from Egypt and Kenya. However, the type C viruses were distinct from but related to the Kenyan vaccine strain (K267/67). Serotype SAT2 topotype outbreaks (SAT2/XIV, SAT2/XIII, and SAT2/IV) were reported in Ethiopia, Sudan, and Kenya, respectively [123].

**Fig. 5:**
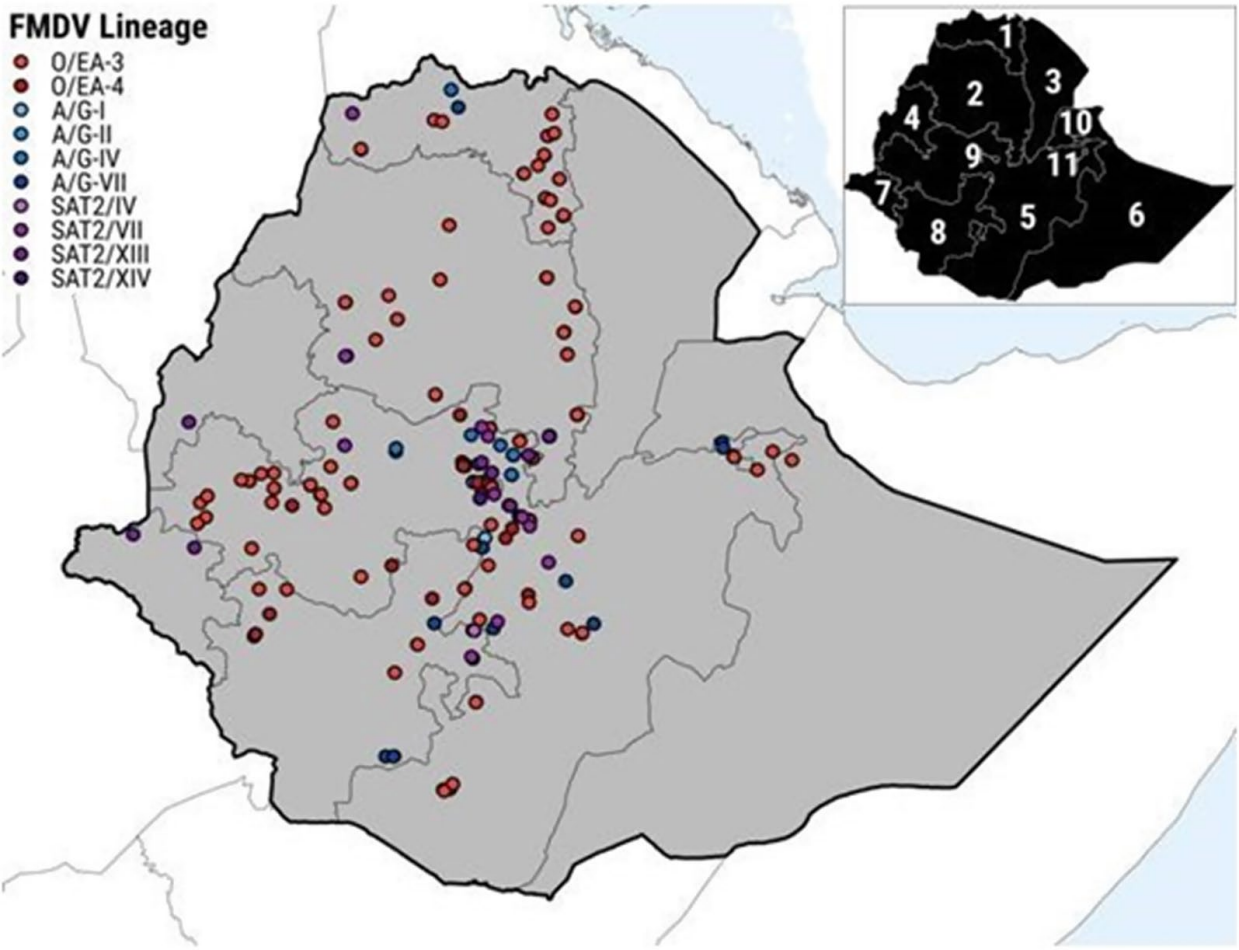
FMDV isolates acquired in Ethiopia from 2008 to 2019 were distributed across the country. The map was adapted from https://onlinelibrary.wiley.com/doi/10.1111/tbed.13675

Phylogenetic analysis of FMDV VP1 coding region sequences from 2008 to 2017 revealed seven different FMD viral clades in Ethiopia: O/East Africa-3 (EA-3), O/East Africa-4 (EA-4), A/AFRICA/G-I, A/AFRICA/G-IV, A/AFRICA/G-VII, SAT2/VII, and SAT2/XIII (Fig. 5) [81]. These strains were related to other East African FMDV strains but also showed some genetic variation within and among serotypes. Molecular characterization of FMDV strains can help monitor the disease epidemiology and select suitable vaccines for FMD control in Ethiopia. However, only FMDV serotype SAT2 (classified into two topotypes: SAT2/VII and SAT2/XIII) was detected for the first time in the three Afar regions in a 12-year retrospective study, while no other FMDV serotypes were detected in this area [15].

Serotype C of FMDV was first detected in Europe in the 1920s and then spread to other continents. It was less prevalent and less severe than other serotypes. The last outbreaks of serotype C occurred in Brazil and Kenya in 2004 [27]. In Ethiopia, serotype C emerged in 1957 along with serotype O [96] and remained endemic until 1983, coexisting with serotypes O, A, and SAT1 [127]. Paton, et al. [27] reported that no cases of FMDV serotype C have been detected globally since 2004. Therefore, they suggest stopping the production, testing, and use of serotype C vaccines and stocks in worldwide repositories. They also advise restricting the in vitro handling of the virus to situations where risk assessment supports it and biosecurity measures are stringent. Furthermore, they encourage researchers to join global monitoring efforts for serotype C [27].

Molecular epidemiology showed that most of the FMDV type O isolates from Egypt, Eritrea, and Sudan belong to the EA-3 topotype. This topotype was also detected in Ethiopia every year from 2008 to 2019, except for 2016. The O/EA-4 topotype was mostly found in the central and southwest regions of Oromia [81]. It was first detected in Ethiopia in 2005 and has largely remained there, with only a few outbreaks in Kenya in 2010. Currently, EA-2 and EA-3 are the main topotypes responsible for most of the serotype O outbreaks in the region [128]. Serotype A was detected sporadically from 2008 to 2019 and belonged to three different genotypes: A/AFRICA/G-I, A/AFRICA/G-IV, and A/AFRICA/G-VII. Serotype SAT2 was also detected intermittently from 2009 to 2018, belonging to two different topotypes: SAT2/VII and SAT2/XIII [81]. The CHY topotype of FMDV serotype O and the Cathay topotype of FMDV serotype Asia 1 have higher evolutionary rates than the other topotypes, which may indicate their adaptation to different hosts and environments [126].

## The economic consequences of FMD

### Global impact of FMD

FMD outbreaks affect the entire cattle agribusiness chain, resulting in (1) a reduced cattle population, (2) increased imports of cattle and beef, (3) decreased beef consumption, and (4) huge economic losses [55, 129]. FMD also has a severe social impact on farmers, who may suffer from illness, stress, depression, stroke, divorce, or even suicide. FMD outbreaks in FMD-free countries or zones trigger strict and costly control measures, such as culling, movement restrictions, and trade bans, until the disease is eradicated, and the FMD-free status is regained. For instance, FMD outbreaks in the United Kingdom, Japan, and South Korea have shown the vulnerability of even advanced biosecurity systems to FMD introduction and spread and the consequent losses in domestic and export markets [132, 134]. FMD also hampers the trade potential of low- and middle-income countries, where most of the world's cattle population resides but only a small fraction of global livestock exports originate. For example, Uganda’s endemic FMD situation prevents it from accessing lucrative export markets [133]. Zambia faces an annual loss of $1.6 billion in exports of beef and sable antelopes due to FMD-related import bans from Botswana and South Africa [134].

FMD control requires cooperation and public investment in veterinary services and surveillance systems within and between countries, which can also help control other livestock diseases. The Global FMD Control Strategy aims to help poor countries and animal trade by enhancing FMD control in endemic regions and safeguarding FMD-free regions [74]. FMD control can reduce the direct effects of the disease on animal production and productivity, which can cost USD 6.5–21 billion a year in endemic regions [73, 135]. It can also mitigate the indirect effects of the disease on market access, technology adoption, food security, income, and livelihoods of livestock-dependent households, particularly in low-income countries [55, 73]. Moreover, FMD control can prevent or minimize export losses due to trade restrictions on livestock and livestock products, which can constitute the majority of the total costs of an FMD outbreak, depending on the country and the control measures implemented [136].

### Economic losses of FMD in Ethiopia

FMD is a widespread and devastating disease in Ethiopia that affects the well-being and income of livestock farmers and other actors in the sector [53]. The disease not only reduces animal productivity by causing lower milk production, reproductive problems, calf mortality, and early culling but also limits market access and tourism opportunities due to trade barriers, movement restrictions, and negative perceptions [49]. The country lost more than 14 million USD as a result of the export ban between 2005 and 2006 [137]. Similarly, an economic loss of 3,322,269 USD was reported in 2011 due to bull export rejection from an international market [95]. The economic losses due to FMD in northwest Ethiopia varied by production system. The mixed crop-livestock (MCL) system had lower losses of USD 34 per affected herd than the commercial dairy farms, which had losses of USD 459.1 per affected farm [49].

The average losses per herd and per animal in the MCL system were 76 USD and 9.8 USD, respectively. In the pastoral system, the average losses per herd and per animal were 174 USD and 5.3 USD, respectively. The animal-level mortality losses were 129 USD in the MCL system and 151 USD in the pastoral system [138]. In addition to export losses and control costs, an estimated annual economic loss of 1,350 million ETB was incurred. However, production losses were the costliest. Furthermore, an average of 40 million ETB and more than 300 million ETB were lost per year due to FMD export costs and animal death, respectively [76].

Tadesse, et al. [49] analyzed the economic losses due to FMD in commercial dairy farms and MCL production systems. They found that the average economic loss per dead animal was USD 194.45, ranging from USD 30.8 in young stock to USD 388.9 in draught ox. The average daily milk loss per affected lactating cow was 1.85 L (L), with 1.4 L in local cows and 2.9 L in crossbreed cows. FMD affected the milk production of cows differently depending on the production system. The average milk loss per infected cow was USD 26 in the mixed crop-livestock (MCL) system and USD 97.5 in commercial dairy farms [49]. They also found that mortality loss, milk loss and draft loss contributed to the economic loss at the animal level in the MCL production system, while milk loss was the main loss in commercial dairy farms, where no mortality loss was observed. This suggests that commercial dairy farms provided better care for infected animals [49]. Hence, future research should aim to develop and evaluate effective control measures to mitigate the morbidity and economic losses resulting from FMD.

FMD is a highly contagious viral disease that affects many kinds of animals, including livestock and wildlife [140]. To control FMD, countries need to assess their disease risk and implement appropriate measures to reduce it. One framework for doing this is the Progressive Control Pathway for FMD (PCP-FMD), which guides countries to gradually and systematically improve their FMD situation [141]. Some of the measures that countries can use to control FMD are destroying infected and exposed animals, restricting animal and animal product movement, and vaccinating susceptible animals. However, these measures have limitations because the FMD virus has many types and can spread easily [139]. Therefore, it is also important to develop better diagnostic and vaccine technologies, strengthen veterinary services and laboratories, coordinate national and regional actions, and educate and motivate farmers, traders, consumers, and policymakers to adopt good practices [79].

## Diagnosis of FMD

### Clinical diagnosis

**Fig. 6:**
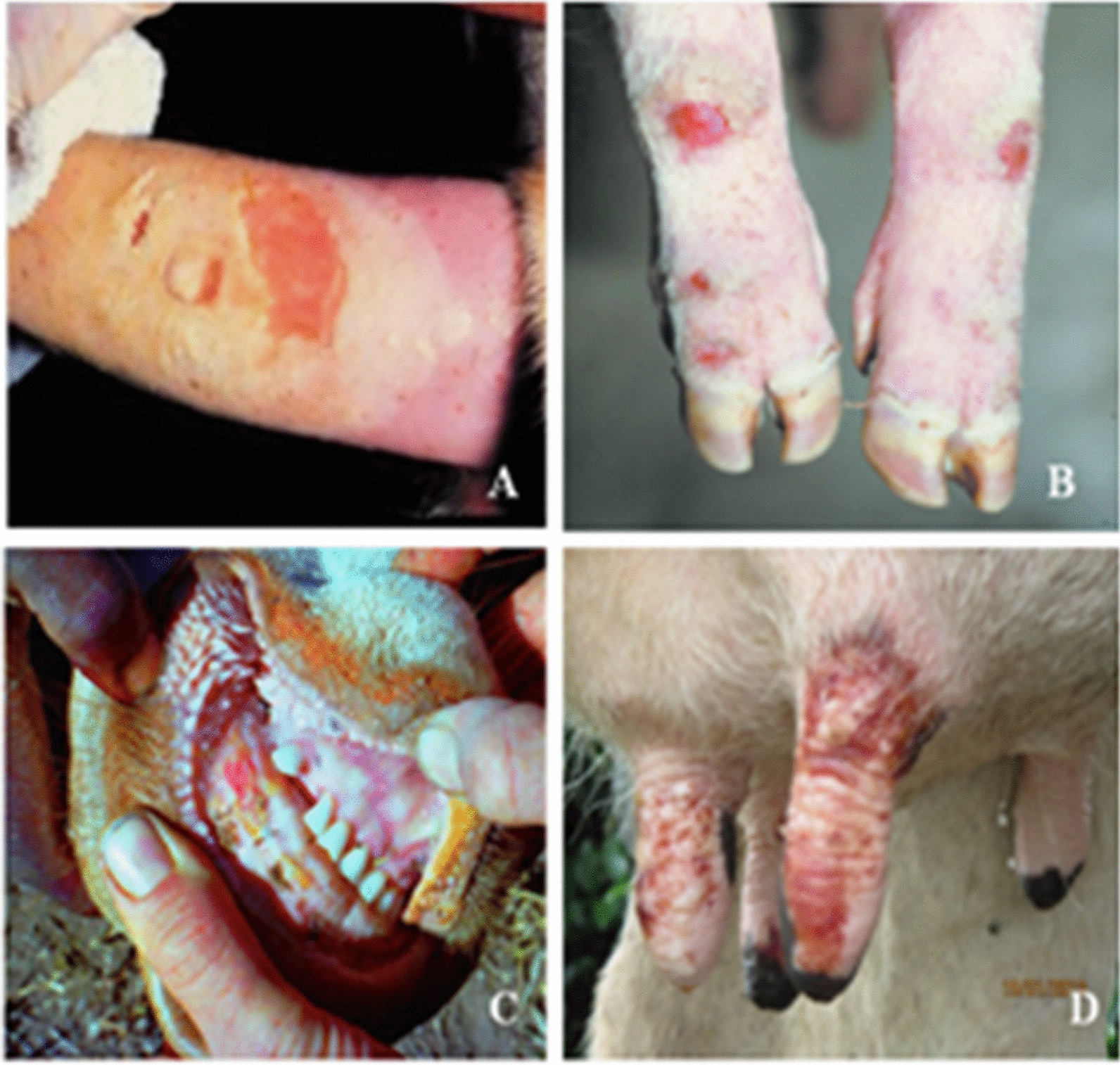
Ruptured blister on a cow's tongue (**A**); foot lesions on the coronet and interdigital area of the hoofs (**B**); affected animal develops vesicles in the muzzle (**C**) and on the teats (in lactating animals) (**D**); source: https://www.cfsph.iastate.edu/diseaseinfo/disease-images/?disease=foot-and-mouth-disease (accessed on 17 July 2023)

FMD is a viral disease that spreads quickly and easily among cloven-hoofed animals, both domestic and wild. The disease can cause fever, loss of appetite, weight loss, lameness, drooling, and depression in infected animals. Blister-like sores on the mouth, teats and hooves are other characteristics of FMD (Fig. 6) [7]. It can also produce blisters on the mouth, feet, and udder that break and heal over time. The hoof wall may show growth rings due to damage to the skin around the hoof. The clinical signs of FMD vary depending on several factors, such as the type of virus, dose of exposure, age and breed of the animal, host species, and immune status. The OIE Terrestrial Manual (chapter 3.1.8) provides a detailed description of how FMD affects different animal species [54].

FMD diagnosis requires laboratory confirmation, as the clinical signs may be mild or unusual in some cases or may resemble other diseases that cause blisters. Laboratory tests can identify the virus or its antibodies in samples from suspected animals. The analysis of the lesion characteristics can also help to determine the source and duration of the virus infection or circulation. FMD diagnosis is important for effective control and surveillance of the disease [142].

### Differential diagnosis

Clinical signs and postmortem findings can indicate FMD, but they are not conclusive because other vesicular diseases [143] can cause similar symptoms. Therefore, other vesicular disorders, such as SVD, vesicular stomatitis, traumatic stomatitis, and vesicular exanthema, should be ruled out when diagnosing FMD. Pigs can develop SVD, vesicular stomatitis, and FMD, while cattle can develop vesicular stomatitis and FMD [65]. Clinical signs are more noticeable in highly susceptible animals; however, in regions where FMD is endemic, clinical signs may be mild or ambiguous due to partial natural immunity or vaccinal immunity [144]*.* Therefore, laboratory tests are essential to confirm any suspicion of FMD and to stop the disease from spreading further [145].

### Laboratory diagnosis

**Fig. 7:**
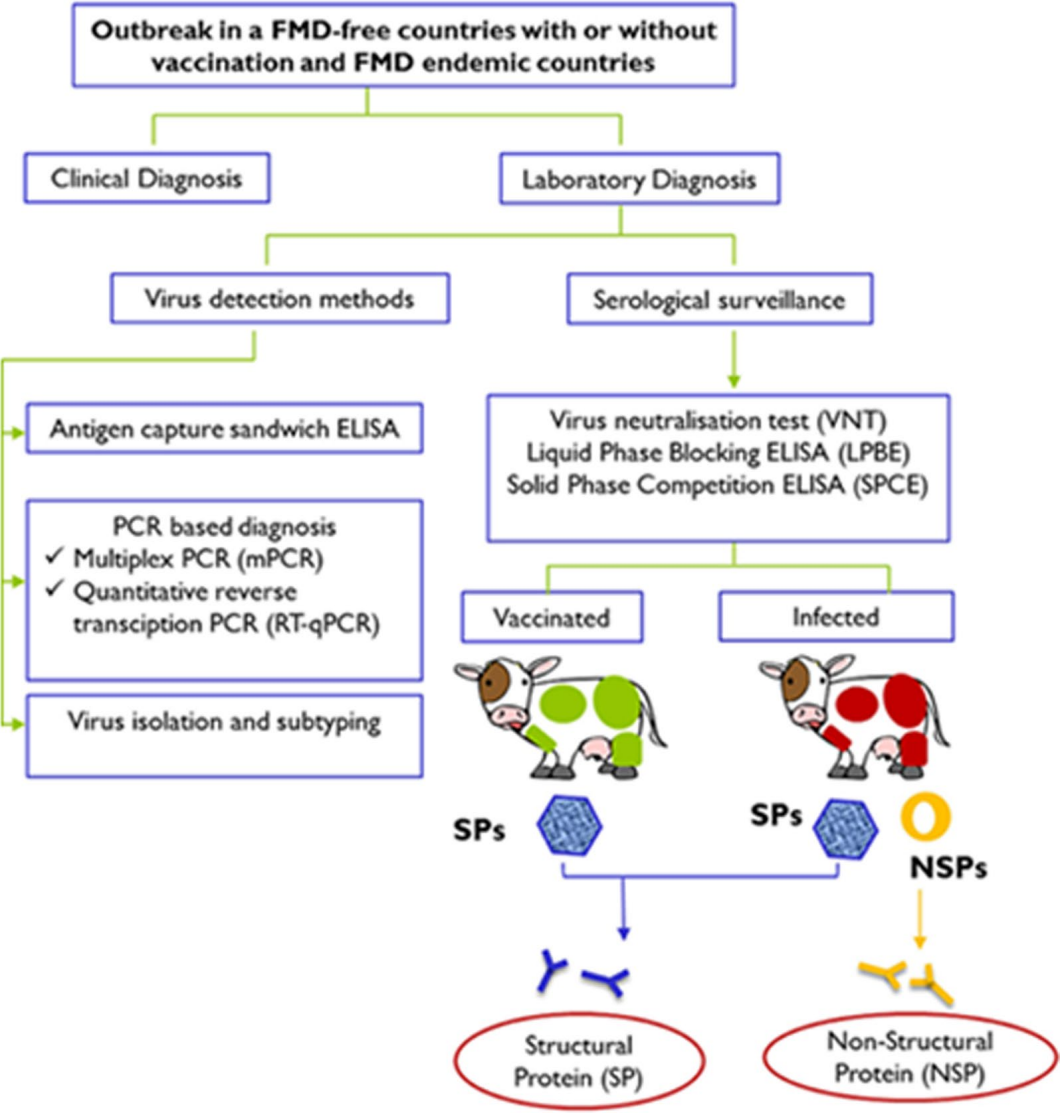
Laboratory tests for FMD diagnosis based on viral materials and antibodies in different scenarios; adapted from https://www.ncbi.nlm.nih.gov/core/lw/2.0/html/tileshop_pmc/tileshop_pmc_inline.html?title=Click on image to zoom&p = PMC3&id = 7473413_fvets-07–00477-g0001.jpg (Accessed on 18 July 2023). Source: [145]

FMD diagnosis is important for effective control and prevention of the disease, as well as for avoiding socioeconomic impacts due to trade barriers imposed by FMD-free countries [145]. This review article provides an overview of some of the current diagnostic methods for FMD, such as ELISA-based methods and molecular methods. FMD diagnosis can be performed by detecting FMD virus or its specific antibodies in various samples from animals with clinical signs or exposure to the virus, such as fluid or tissue from blisters, fluid from the throat, milk, blood, or saliva [54]. Traditional methods of FMD diagnosis include virus isolation (VI), which is the gold standard but is slow and labor intensive and requires high-level biosafety facilities [146]; the virus neutralization test (VNT), which is highly specific and sensitive but also requires live virus and cell culture facilities [147]; the complement fixation test (CFT); and ELISA. In response to an FMD outbreak, prompt actions are typically implemented to ascertain a differential and definitive diagnosis, which is crucial to impede further dissemination of the disease (Fig. 7). Moreover, in conjunction with vaccination and stamping out policies, prompt FMD detection in cloven-hoofed animals utilizing currently available diagnostic instruments has been extensively employed as a strategy to combat this highly scrutinized agent [145].

The complement fixation test (CFT) is a serological method that measures antibodies against FMD virus in serum samples. It is easy and inexpensive, but it has low sensitivity and specificity and can be affected by factors that disrupt complement activity in the samples [148]. Enzyme-linked immunosorbent assay (ELISA) is a common method for FMD diagnosis that can detect either FMD virus antigens or antibodies in different samples. ELISA has several benefits [145]. However, molecular methods have some limitations, such as the need for RNA extraction, the risk of false-positive results due to contamination or nonspecific amplification, and the inability to indicate whether the detected RNA is associated with infectious virus [149]. Molecular methods for FMD diagnosis are based on detecting the genetic material of FMD virus in samples. Some of these methods are conventional PCR, multiplex PCR (m-PCR), real-time PCR (RT‒qPCR), reverse transcription loop-mediated isothermal amplification (RT-LAMP), reverse transcription recombinase polymerase amplification (RT-RPA), reverse transcription insulated isothermal PCR (RT-iiPCR), and reverse transcription droplet digital PCR (RT-ddPCR) [145].

Inactivated vaccines for FMD contain purified FMDV antigen that lacks most of the viral nonstructural proteins (NSPs). These vaccines stimulate antibodies mainly against the viral proteins that form the structure (SP), which help the virus attach and enter the cells. However, when animals are naturally infected with FMDV, they produce antibodies against both SP and NSP, which play a role in virus replication and pathogenesis. Therefore, tests that can detect antibodies against NSPs can distinguish animals that are infected from those that are vaccinated (DIVA test) [150]. DIVA is an important strategy for FMD control and eradication programs that use vaccination as a tool. One of the most widely used NSPs for DIVA testing is the 3ABC polyprotein, which is highly conserved and immunogenic across all FMDV serotypes [151, 152].

Several methods based on ELISA can detect antibodies to the 3ABC polyprotein, a nonstructural protein of FMDV, in serum samples from various animals. These methods include liquid-phase blocking (LPBE-3ABC), solid-phase competition (SPC-3ABC), and direct or indirect sandwich ELISA (ELISA-3ABC). They have high sensitivity and specificity for FMD diagnosis. Immunoblotting can also detect nonstructural proteins and confirm the ELISA results. It can also show which nonstructural proteins are recognized by the antibodies. NSPs can help to monitor the FMDV infection status and vaccination efficacy in endemic regions with active vaccination programs [150]. In Ethiopia, FMD diagnosis is performed by using virus isolation, 3ABC-ELISA, virus neutralization tests, and conventional PCR at the National Veterinary Institute (NVI) and the National Animal Health Diagnostic Center (NAHDIC).

## Strategies for control, prevention, and treatment of FMDV

Foot-and-mouth disease (FMD) prevention and control require a lot of resources and efforts, which are mostly provided by low- and lower-middle-income countries. FMD has seven different strains, each of which needs a specific vaccine. Therefore, FMD prevention depends on the ability to detect the disease early, to warn other countries and regions, and to monitor the animal populations for signs of infection [7]. FMDV infection can be prolonged by antibiotics, but they do not clear the virus from the body. Antibiotics can help infected animals survive longer, but they also increase the risk of virus persistence and transmission. The virus can stay in the pharyngeal epithelia of some animals for a long time, making them carriers of the disease. These carriers can infect other animals through their saliva and nasal secretions, either directly or indirectly [34, 153]. Different countries have different strategies to control FMD, depending on their resources and regulations. In developed countries, the main strategy is to cull and dispose of infected or exposed animals and disinfect their premises. This can prevent the spread of the disease and protect the trade of animal products [154, 155].

However, in Ethiopia, where livestock is a vital source of income and food security, culling is not feasible. Instead, infected animals are isolated and treated with supportive care, such as antibiotics, anti-inflammatory drugs, and wound care. This can reduce the death and suffering of the animals, but it does not stop the virus from persisting and transmitting [156]. This allows the virus to remain in the animal population and cause recurrent outbreaks of FMD [157]. Antibiotic maintenance may have multiple impacts on FMD transmission, which are not well understood or documented. It may alter the gut microbiota of animals, which may impair their immune system and increase their susceptibility to FMD infection [158]. It may also promote the development and spread of antibiotic resistance genes, which may be transferred to other microorganisms through horizontal gene transfer. This may lead to the emergence of novel or more virulent FMD virus strains [159].

Furthermore, it may enable the movement and trade of animals across regions or borders, which may expose them to different sources and types of FMD virus. These potential effects of antibiotic maintenance on FMD transmission pose serious challenges and risks for animal health, public health, and food security in low-income countries [160]. Therefore, it is important to promote the rational use of antibiotics and implement appropriate biosecurity measures to prevent and control FMD in low-income countries [161]. We also need more research and evidence to understand how and why these effects happen, and what we can do to stop them. This also requires a One Health approach that considers the health of animals, humans, and the environment together.

### Vaccination

FMD control strategies and vaccine development vary by disease status of the country or region. In FMD-free countries, the main strategy is to stamp out infected and in-contact animals, disinfect premises, and vaccinate animals around outbreak areas [61]. In endemic countries, these options are not feasible. Hence, following the OIE/FAO progressive control pathway helps to enhance veterinary services, outbreak preparedness and vaccination programs [68]. In many developing countries, vaccination with an inactivated whole-virus vaccine is the only way to control the epidemic [162]. Some countries or regions have eliminated FMD by using inactivated FMD vaccines as part of mandatory vaccination programs. However, FMD remains endemic in parts of Africa and Asia [6]. One of the limitations of inactivated FMD vaccines is that they confer short-term immunity and require frequent boosting [162].

**Fig. 8:**
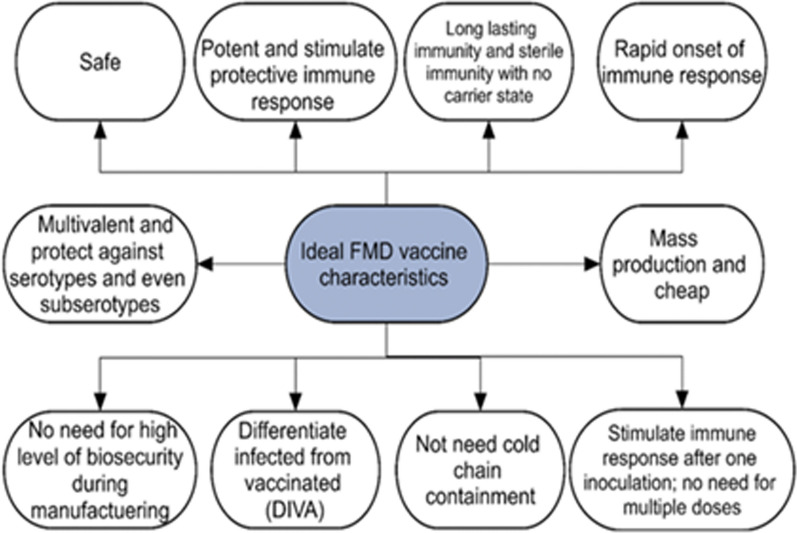
Desirable features of a perfect FMD vaccine; Adapted from https://link.springer.com/article/10.1007/s00705-019-04216-x/figures/2 (Accessed on 17 August 2023). Source: source [139]

FMD virus infects a wide range of wild and domesticated cloven-footed mammals and can spread rapidly across borders. FMD outbreaks can have severe economic consequences for the livestock industry due to trade restrictions and reduced animal productivity. An ideal vaccine formulation should meet the following criteria: safety, thermal stability, low cost, multivalency, rapid and long-lasting immunity with a single dose, and compatibility with DIVA (differentiating infected from vaccinated animals) principles (Fig. 8) [139]*.*

Currently, inactivated vaccines are the most widely used to prevent FMD in endemic regions, but they have several limitations, such as the need for high-containment facilities to grow virulent FMDV, the limited cross-protection among different strains and topotypes within the same serotype, the frequent emergence of new variants that may escape vaccine-induced immunity, and the inability to eliminate virus carriers [68].

FMD vaccines mainly consist of inactivated viruses that elicit protective antibodies against virus structural proteins. However, when FMDV infects an animal, it also reproduces and makes the body produce more antibodies against its inner proteins. These antibodies can help to distinguish.

animals that are infected from those that are vaccinated (DIVA), which is important for knowing how widespread or rare the infection is. Additionally, NSP antibody tests can identify FMD infection regardless of the virus serotypes present [150, 164]. However, some new FMDV strains found in Egypt could not be identified by the usual molecular methods for serotyping, suggesting that they are different from the ones that the current FMDV vaccines can protect against [122].

A recent study by [11] reported a high seroprevalence of FMDV and some risk factors (district, age, body condition score, and management system) in Ethiopia, suggesting the need for tailored control and prevention strategies based on the circulating virus serotype. However, determining the circulating virus serotype is difficult in Ethiopia, as FMD outbreaks caused by serotypes A, O, and SAT2 have occurred in different regions [99].The current trivalent inactivated vaccine used in Ethiopia for FMDV serotypes O, A, and SAT2 contains vaccine strains ETH/38/2005, ETH/6/2000, and ETH/64/2009, respectively [165]. These vaccine strains may not match well with the field strains and may provide suboptimal protection. Outbreak reports guide FMD control measures in the country. However, few private livestock owners vaccinate their animals regularly or before FMD outbreaks due to various reasons, such as lack of awareness, cost, or availability of vaccines. Continuous molecular monitoring of circulating FMDV strains is strongly recommended to ensure the selection of the appropriate strain for the preparation of effective vaccines [14]. According to a vaccine-matching study conducted in Ethiopia, the O/ETH/38/2005 vaccine strain can provide protection against outbreaks caused by the O/EA-3 topotype but has lower efficacy for the O/EA-4 topotype [166].

Additionally, the amount of FMD vaccines produced in Ethiopia is insufficient compared to the country’s livestock population, and the vaccines are also expensive, making livestock owners reluctant to pay for them [167]. To reduce the economic losses caused by FMD, the government should implement policies that include vaccination and movement restrictions for livestock. These actions can stop FMD from spreading in the country, which can happen because of poor or missing vaccination, free animal movement, and the market chain[99]. Additionally, not knowing enough about the molecular details of FMD makes it harder to control it [81]. Therefore, a molecular understanding of virus-antibody interactions is required for the development of better vaccines as well as timely assessment of the spread and severity of epidemics [168].

The vaccine development efforts against FMD are not commercially attractive despite the huge market potential because of various technical, regulatory, and economic challenges [169]. Some of these challenges include the costly biosafety facilities required to produce live virus, the need to differentiate infected from vaccinated animals, and the variable local regulatory restrictions to produce and commercialize the vaccine. The high investment and operational costs of FMD vaccine production and delivery, the low profit margin and market share of FMD vaccines, the lack of incentives and subsidies for FMD vaccine research and development, and the competition and conflicts of interest among different stakeholders in FMD control and eradication also hinder the development of novel vaccines that overcome these limitations and provide effective and safe protection against FMD [153, 170–172].

### Application of nanoliposomes

Although commercially available FMD vaccines are effective, they provide short-term immunity requiring regular boosters. A new FMD vaccine is needed to improve immunization, safety, and long-term immune responses. A synthetic peptide vaccine is one of the safe and important vaccines. Peptide vaccine has low immunogenicity, requiring strong adjuvants. Nanoliposomes can be used as new adjuvants to improve immune response. In the current study, nanoliposomal carriers were selected using Dimyristoylphosphatidylcholine (DMPC), dimyristoyl phosphoglycerol (DMPG), and Cholesterol (Chol) as an adjuvant containing two immunodominant synthetic FMDV peptides. The liposomal formulations were characterized by various physicochemical properties. The size, zeta potential, and encapsulation efficiency were optimized, and the obtained nanoliposome was suitable as a vaccine [162].

Nanoliposomes are tiny vesicles made of lipids that can carry drugs or antigens inside them. They are promising candidates for delivering vaccines against FMD. Nanoliposomes can enhance the immune response to FMD vaccines by protecting the antigens from degradation and targeting them to specific cells. Nanoliposomes can also release antigens slowly and steadily, which may improve the duration and quality of immunity. Lycium barbarum polysaccharides (LBP) are natural compounds that have immunomodulatory effects. They can be encapsulated in nanoliposomes to act as adjuvants for FMD vaccines. LBP-nanoliposomes have been shown to induce higher antibody levels and stronger cellular immunity than conventional adjuvants in mice [173]. Therefore, nanoliposomes are a potential nanotechnology platform for developing more effective and safer FMD vaccines. However, vaccines take several days to induce a response and need a booster dose to sustain herd immunity [174].

### Surveillance and biosecurity

The main source of FMD surveillance data in Ethiopia is the passive reporting system, where farmers and veterinarians notify the National Animal Health Diagnostic and Investigation Center (NAHDIC) and the National Veterinary Institute (NVI) of suspected FMD cases. These centers also perform laboratory confirmation and molecular typing of FMDV isolates obtained from outbreak investigations [81, 175]. To prevent and control FMD transmission among livestock, Ethiopia implements various biosecurity measures at different levels, such as restricting animal movements, enforcing quarantine regulations, conducting vaccination campaigns, applying disinfection protocols, disposing of infected carcasses and animal products safely, and raising public awareness and education on FMD prevention [12, 175]. However, these measures may not be very effective in Ethiopia due to several challenges, such as the coexistence of multiple serotypes and subtypes of FMDV, free livestock movement, scarcity of effective and affordable FMD vaccines, lack of regular vaccination campaigns, and inadequate public awareness of FMD prevention.

FMD surveillance and biosecurity in Ethiopia face several challenges, such as lack of adequate resources, infrastructure, personnel, coordination, and data management; low coverage and efficacy of vaccination; high diversity and mobility of livestock; cross-border transmission of FMDV; and socio-economic factors that influence farmers’ compliance and participation. Moreover, FMD surveillance and biosecurity in Ethiopia also offer some opportunities for improvement, such as enhancing the capacity and collaboration of national and regional laboratories; applying novel diagnostic and epidemiological tools; developing effective and tailored vaccines; engaging stakeholders and communities; and harmonizing regional and international efforts to control FMD [12, 81, 175].

FMD control and surveillance require prompt and accurate disease confirmation [176]. FMD can also be controlled by restricting animal movement, isolating infected animals, and disinfecting infected premises [177, 178]. However, FMD vaccine development and selection are challenging due to the antigenic diversity and genetic variation of FMDV serotypes and strains [179]. Antibodies induced by one serotype do not protect against other serotypes and may not cross-protect within the same serotype [80]. Therefore, it is essential to monitor new FMDV strains to prevent their spread and ensure effective vaccination programs. The identification of new FMDV variants will help to update vaccine production and control measures [8, 122]. FMD vaccines are often designed to cover multiple serotypes and strains, but this compromises their efficacy and cost compared to monovalent vaccines [180], making the use of vaccination to control FMD challenging for developing countries with limited resources such as Ethiopia [167].

Epidemiological techniques such as routine disease data collection in the population (monitoring and surveillance) are essential for detecting newly emerged serotypes for vaccine matching studies, which help to maintain effective vaccine production [166]. In Ethiopia, where FMD is endemic, new viral strains may emerge. Hence, the surveillance system needs to implement several actions, such as notifying suspected cases by farmers and veterinarians, investigating suspected and high-risk properties, conducting serological surveys, and monitoring animals in slaughterhouses. The authors also recommend more FMD surveillance and multivalent vaccines and emphasize regular vaccination and control measures for sustainable food security and rural development.

## Conclusions

FMD is a major threat to the livestock sector in Ethiopia. The virus, which has multiple serotypes and strains, spreads through transboundary animal movement. Due to the high genetic variation and antigenic diversity of FMDV, infection with one genetic lineage does not provide full protection against another lineage in the same serotype. Different lineages of the same serotype that evolved independently in different geographical regions are called different topotypes, and the current trivalent vaccines in Ethiopia may not cover all of them. FMD affects the domestic and international markets of livestock products, as well as food security and economic development in the country. Therefore, effective control measures are needed to combat FMD. We propose the following recommendations:

- Develop better and multivalent vaccines that provide longer and wider protection against different FMDV strains.
- Regulate cattle movement and prevent contact between infected and susceptible herds.
- Ensure adequate vaccine supplies to endemic areas and encourage regular vaccination among livestock owners.
- Monitor the antigenic diversity and evolution of FMDV in the country through vaccine matching studies.
- Understand the spatial and temporal distribution of FMD at the national level through comprehensive surveillance studies.

## Data Availability

Not applicable.

## Abbreviations

Abs: Antibodies
CFT: Complement fixation test
DIVA: Differentiate infected from vaccinated animals
EA: East Africa
ELISA: Enzyme-linked immunosorbent assay
ETB: Ethiopian Birr
FMD: Foot-and-mouth disease
LPBE: Liquid-phase blocking ELISA
L^pro^: Leader proteinase
MCL: Mixed crop-livestock
m-PCR: Multiplex-PCR
NAHDIC: National animal health diagnostic and investigation center
NSP: Nonstructural protein
NVI: National Veterinary institute
ORF: Open reading frame
PCR: Polymerase chain reaction
RNA: Ribonucleic acid
SAT: South African territories
SP: Structural protein
SVD: Swine vesicular disease
USD: United States dollar
VNT: Virus neutralization test
VP: Viral protein
